# Comprehensive multivariate insights into external training load in women’s football: four session clusters to optimize weekly periodization

**DOI:** 10.5114/biolsport.2026.154943

**Published:** 2025-11-21

**Authors:** Eider Barba, Beñat Erkizia, David Casamichana, Udane Etxebeste, Julen Castellano

**Affiliations:** 1 Real Sociedad Institute, Real Sociedad S.A.D., Donostia-San Sebastián, Spain; 2 University of the Basque Country (UPV/EHU), research group GIKAFIT, Vitoria-Gasteiz, Spain

**Keywords:** Female, GPS, Team sport, Soccer, Microcycle, Monitoring

## Abstract

This study aimed to analyse the external load demands placed on female football players during training sessions and matches across different competition levels. Twelve external load metrics were monitored in 58 players from professional (n=19, PRO), reserve (n=18, RES), and under-17 (n=21, U17) teams across 251 training sessions and 85 matches. Data were collected with GPS devices and analysed using principal component analysis (PCA, Varimax rotation) and K-means clustering (K = 4). PCA grouped the metrics into three components: high-speed movements, volume (e.g., duration, total distance, acceleration load, decelerations > 3 m · s^−2^), and speed-change variables (accelerations and decelerations). Together, these three components explained approximately 90% of the total variance. Cluster analysis identified four session types: (1) low-demand sessions (introductory, pre-match, and discontinuous training), (2) high-speed but moderate-demand sessions (compensatory and extensive training), (3) highest-demand sessions (official matches), and (4) high-neuromuscular-demand sessions (intensive training). Clusters 2 and 3 showed the most significant differences in variables such as high-speed distances and maximum speed, highlighting distinct physical demands. Except for the U17 team in intensive sessions and matches and PRO in discontinuous sessions, all teams distributed their sessions in nearly consistent patterns across clusters. The study underscores the importance of integrating external load variables to profile training demands and demonstrates the value of cluster analysis for optimizing training planning and periodization. Practically, the four clusters provide simple guidelines for coaches to balance speed, volume, and neuromuscular demands within weekly training.

## INTRODUCTION

In recent years, there has been growing interest in understanding the specific demands of training in women’s football [1, 2], as the sport continues to grow both in popularity and professionalism [3]. Despite the similarities in game structure and training methodology between men’s and women’s football, it is increasingly recognized that female players face unique physical, tactical, and physiological challenges that require tailored load management approaches [4, 5]. Therefore, although findings from men’s football provide valuable reference points, they cannot be directly extrapolated, which underlines the pressing need for more comprehensive studies specifically focused on women’s football [6].

In men’s football, studies have shown that the heaviest training loads are typically scheduled in the middle of the microcycle, with a gradual reduction in workload as matchday approaches [7]. Sessions 4 days before the match day (MD-4) and MD-3 have been reported to emphasize accelerations, decelerations, and sprinting, while MD-2 and MD-1 sessions often involve tapering, with a focus on lower-intensity activities designed to optimize recovery before the competition [7, 8]. However, the transferability of these findings to women’s football remains unclear, as the physical responses to training stimuli can differ significantly between male and female athletes. For instance, factors such as differences in muscle mass [9] and aerobic capacity [10] might influence the optimal structuring of training loads for female players. Although the menstrual cycle has often been considered a determinant of performance [11], recent reviews highlight that its influence is not consistent: some evidence suggests potential effects [12], whereas others report no significant influence on acute strength performance or adaptations to resistance training [13]. This factor should therefore be treated with caution in women’s training design.

Additionally, tactical periodization—another common approach for categorizing football training sessions—has typically classified sessions based on the predominant type of effort (e.g., tension, endurance, or speed) [14, 15]. Although widely applied in men’s football, there is limited evidence to suggest how this method works in women’s football [16]. In women’s teams, where the physiological responses to load and recovery patterns might be distinct, it is crucial to determine whether the same periodization principles hold true. Recent studies in male athletes have shown that internal load indicators, such as salivary and biochemical markers, reveal distinct physiological perturbations in response to varying training loads [17, 18]. Similar investigations in women’s football are necessary to identify their unique responses and optimize training prescriptions. For example, female players may have different recovery needs, and training designs may need to account for gender-specific factors, such as increased susceptibility to anterior cruciate ligament (ACL) injuries and other biomechanical considerations [5, 19].

One of the main gaps in the current literature is the lack of detailed studies examining how training load is distributed across teams within a professional women’s football club, particularly from a multivariate perspective. There is growing recognition that multivariate analysis, such as principal component analysis (PCA) and cluster analysis, can offer valuable insights into the external training load, especially in complex team sports environments such as football [20]. These techniques have been successfully applied to male football players [21], enabling researchers to reduce large datasets and identify key external load variables of training tasks [21] and training sessions [22] or styles of play in official matches [23]. However, few studies have applied these methods in elite women’s football, leaving a significant knowledge gap. Cluster analysis is especially relevant because it translates large datasets into practical session typologies, which coaches can easily apply to structure weekly training [21, 22].

Given the increasing competitiveness and professionalization of women’s football, understanding the specific demands placed on female athletes is more critical than ever [24]. By applying multivariate approaches, this study aims to provide a comprehensive analysis of the external training load in elite women’s football, focusing on how external workload is distributed across different types of sessions throughout the microcycle. This approach will allow us to explore the unique physical demands female players face and assess whether current training structures are optimized for their development and performance. Additionally, this research will investigate the coherence of training methods across different teams within the same professional women’s football club, ensuring that the transition between age groups or developmental stages is aligned with the physical demands of PRO team football. Based on this rationale, we hypothesize that external training load in women’s football can be summarized into a reduced number of key components and grouped into distinct session clusters, which align with competition demands and provide a framework for optimizing weekly periodization.

## MATERIALS AND METHODS

### Subjects

Fifty-eight female soccer players from different age categories of the same professional Spanish club were included in this study: professional first team (PRO; n = 19; age: 23.1 ± 2.8 years; stature: 166.4 ± 6.2 cm; body mass: 61.8 ± 5.3 kg), reserve team (RES; n = 18; age: 19.3 ± 2.2 years; stature: 165.8 ± 5.5 cm; body mass: 59.4 ± 4.6 kg), under-17 (U17) team (U17; n = 21; age: 16.3 ± 1.3 years; stature: 166.6 ± 4.1 cm; body mass: 59.0 ± 6.2 kg). The professional team was playing in the Spanish Women’s First Division (Liga F).

Data were collected as part of the professional team’s daily routines throughout the season. This study adhered to the Declaration of Helsinki, and players provided informed consent prior to their participation. Furthermore, players’ identities were anonymized. Finally, the institutional ethics committee of the university (CEISH) approved the study (code: M10-2024–124).

Inclusion criteria were being an officially registered player of the club and having fully completed a training session or official match during the study period. Exclusion criteria were partial participation in a session or match, injuries that prevented training, or missing GPS data. However, compensatory training sessions designed for players with reduced match exposure were included, as they represented a key strategy to equilibrate weekly load distribution.

### Measures

External load demands were monitored using global positioning system (GPS) devices. A total of 12 external load variables were measured during both training sessions and official matches (OM). These variables included the total duration (DUR, in minutes), total distance covered (TD, in metres), running distance at > 14 km · h^−1^ (TD14, in metres), high-speed running distance at > 18 km · h^−1^ (TD18, in metres), sprinting distance at > 21 km · h^−1^ (TD21, in metres), high sprinting distance at > 24 km · h^−1^ (TD24, in metres), maximum speed (Vmax, in km · h^−1^), acceleration load (aLoad, in arbitrary units, au), number of accelerations at > 3 m · s^−2^ (ACC3) and at > 4 m · s^−2^ (ACC4), as well as decelerations at < -3 m · s^−2^ (DEC3) and at < -4 m · s^−2^ (DEC4), both measured as counts.

The aLoad variable represents a cumulative value where all accelerations and decelerations are treated as positive, offering insight into the overall acceleration demands on the athlete, regardless of their velocity. Previous research has indicated an inter-unit coefficient of variation of 2–3% for aLoad [25], which is lower than the variability typically observed between devices using traditional acceleration detection methods [25].

### Procedures

The research was conducted during the 2023–2024 competitive season. Data on external training load and match performance were collected using GPS units (WIMU SPRO V.980, Almería, Spain) sampling at 10 Hz. Players wore the GPS devices from the beginning of the warm-up until the end of each session or match. The GPS units were secured on the upper back, between the shoulder blades, using a neoprene harness specifically designed to fit each player. After each session, the data were transferred to a computer and analysed using SPRO software. In total, GPS data from 251 training sessions and 85 OM were processed (n = 336), with the following breakdown per team (Table 1): PRO = 125 sessions (1,834 individual GPS files, IGF), RES = 120 sessions (1,609 IGF), and U17 = 91 sessions (739 IGF).

**TABLE 1:** Structure of the typical microcycle for each team, including the number of sessions and matches analysed.

	Monday	Tuesday	Wednesday	Thursday	Friday	Saturday	Sunday	Total
PRO	COM/INTRO	*OFF*	*INT*	*EXT*	*DIS*	*PRE*	*OM*	125
	7/14	-	16	17	22	21	28	

RES	*COM/INTRO*	*OFF*	*INT*	*EXT*	*DIS*	*OM*	*OFF*	120
	12/12	-	24	23	22	27	-	

U17	*OFF/INTRO*	INT	OFF	*EXT*	*DIS*	*OM*	*OFF*	91
	12	5	-	21	23	30	-	

Note: PRO = professional team, RES = reserve team, U17 = under-17 team. INTRO = introductory session, EXT = extensive session, INT = intensive session, OM = official match, DIS = discontinuous session, PRE = pre-match session, COMP = compensatory session.

The weekly training structure of the teams showed some differences, as outlined in Table 1. The day off for the first two teams (PRO and RES) was typically on Tuesday, whereas for the U17 team, it was on Wednesday. Additionally, a pre-match session was exclusive to the PRO team, while for the other two teams, the MD-1 session was a discontinuous session. Due to differences in match schedules—primarily OM on Sundays for the PRO team and Saturdays for the non-professional teams—the PRO team conducted its extensive session on MD-3, while the other teams held theirs on MD-2. Furthermore, the U17 team trained only one Monday every two weeks, with the session being primarily introductory, as their matches usually took place on Saturdays. The structure of the Monday session for the RES team depended on the timing of the previous match: if the match was on a Saturday, the Monday session was introductory, whereas if the match was on a Sunday, the Monday session followed a compensatory structure.

The following training sessions consisted of integrated content, combining tactical, technical, and physical elements:

– Compensatory (COM) session: This session was conducted the day after a match (MD+1) or two days later (MD+2) and divided players into two groups. The first group included players who had participated for 60 minutes or more in the previous match, focusing on recovery with low-intensity activities and regeneration exercises. This group was excluded from the analysis. The second group, comprising players with fewer match minutes (typically less than 45 minutes), engaged in technical drills followed by positional games, small-sided games (SSGs) with goalkeepers (30–60 m^2^ per player), and high-speed or maximumspeed exercises to replicate match demands. This session is “compensatory” because it aimed to equalize the physical workload of those who had played fewer minutes.
– Intensive (INT) session: typically held four days before the next match (MD-4); this session was designed to develop strength and power. It involved small groups of players training in reduced pitch areas. Common exercises included possession or positional games and SSGs with goalkeepers, usually within 25–50 m^2^ per player.
– Extensive (EXT) session: usually conducted three days before the match (MD-3), this session’s objective was tactical preparation for the upcoming game. Activities included large positional games (70–100 m^2^ per player) and 11 vs. 11 match simulations (with areas exceeding 200 m^2^ per player). This session also incorporated maximum speed drills and endurance work through intermittent running.
– Discontinuous (DIS) session: primarily performed two days before the match (MD-2), this session focused on technical and tactical preparation. Typical activities included control and passing drills and tactical exercises.
– Pre-match (PRE) session: This session, conducted the day before a match (MD-1), aimed to activate players for competition, emphasizing agility drills and tactical scenarios that mimicked match situations.
– Official match (OM): The official matches analysed adhered to 11-a-side football regulations, including standard pitch dimensions, goal sizes, player numbers, and game rules specific to the competition in which each team participated.

Table 1 provides an overview of the most common microcycle structures for each team, illustrating the scheduling of each type of training session throughout the week.

### Statistical analysis

First, a descriptive analysis of the external variables analysed for each type of session was conducted. Subsequently, PCA was performed for external training load variables following established methodologies [26]. Prior to conducting the PCA, the suitability of the external training load datasets was assessed using a correlation matrix, Bartlett’s test of sphericity, and Kaiser-Meyer-Olkin (KMO) statistics. Bartlett’s test of sphericity was significant (p < 0.01), and all variables had a KMO value above 0.5, indicating that the data were appropriate for PCA [26]. Components with eigenvalues less than 1 were excluded from extraction [27]. PCA was applied with a VariMax rotation to identify uncorrelated components [22, 28]. This rotation aimed to enhance interpretability by simplifying the component loadings. As a result, each principal component (PC) provided distinct information. The calculation steps for PCA mirrored those employed in previous research [29]. For each extracted PC, only original variables with a loading greater than 0.7 were considered indicative of a strong relationship with the PC [22, 28].

Subsequently, K-means cluster analysis was performed using Jamovi software, with the optimal number of clusters set to K = 4. K-means clustering was employed to identify groups of observations within the multivariate data [30]. Differences between clusters were examined using one-way ANOVA, followed by Duncan’s post hoc test. The magnitude of Cohen’s d effect sizes was interpreted according to the following thresholds [31]: trivial (< 0.2), small (0.2–0.6), moderate (0.6–1.2), large (1.2–2.0) and very large (> 2.0). Following cluster creation, chi-square analysis was conducted to assess the association between clusters and training sessions, both for all teams combined and for each team individually. All statistical analyses were performed using Jamovi version 2.4.14, with significance set at p < 0.05.

## RESULTS

Table 2 shows the average values and standard deviation (± SD) of the external training and match load variables for each of the teams analysed.

**TABLE 2:** Mean ± SD of the external training and match load variables for each of the teams.

External variables	PRO	RES	U17
DUR (min)	65.7 (± 17.7)	69.8 (± 14.7)	73.2 (± 16.3)
aLoad (au)	1,948.0 (± 619.4)	2,189.0 (± 470.3)	2,105.0 (± 587.5)
TD (m)	5,807.0 (± 2,431.0)	6,231.0 (± 2,115.7)	6,496.0 (± 2,542.0)
TD14 (m)	1,024.0 (± 615.6)	912.1 (± 545.3)	1,025.6 (± 505.5)
TD18 (m)	329.9 (± 236.5)	286.0 (± 211.5)	313.2 (± 180.5)
TD21 (m)	158.3 (± 136.1)	132.5 (± 120.4)	154.9 (± 98.7)
TD24 (m)	46.6 (± 51.4)	34.0 (± 36.2)	48.1 (± 45.0)
Vmax (km · h^−1^)	24.8 (± 1.9)	24.6 (± 2.1)	25.2 (± 2.1)
ACC3 (n)	30.4 (± 10.8)	33.3 (± 6.7)	26.7 (± 6.5)
DEC3 (n)	42.3 (± 14.6)	47.3 (± 10.5)	35.6 (± 11.2)
ACC4 (n)	4.2 (± 2.9)	3.7 (± 1.7)	3.0 (± 1.6)
DEC4 (n)	11.1 (± 5.1)	11.2 (± 3.8)	7.3 (± 3.1)

Note: PRO = professional team, RES = reserve team and U17 = under-17 team. DUR = session duration, TD = total distance, TD14 = running distance at > 14 km · h^−1^, TD18 = high-speed running distance at > 18 km · h^−1^, TD21 = sprinting distance at > 21 km · h^−1^, TD24 = high sprinting distance at > 24 km · h^−1^, aLoad = acceleration load (arbitrary units, au), Vmax = maximum speed (in km · h^−1^), ACC3 = accelerations > 3 m · s^−2^ (counts), ACC4 = accelerations > 4 m · s^−2^ (counts), DEC3 = decelerations < -3 m · s^−2^ (counts), DEC4 = decelerations < -4 m · s^−2^ (counts).

Table 3 details the PCA results for the external load variables. The three PCs accumulated similar variance at > 30% (≈35% and ≈32%) for the first two PCs and ≈22% for PC3. The three components accounted for 90% of the variance explained. It can be concluded that the first of the components is represented by the variables most associated with high-speed movement and sprinting, PC2 is represented by the variables associated with the amount of training or volume (DUR, TD, aLoad and DEC3) and PC3 is represented by the variables associated with changes in speed (accelerations and decelerations), with the greatest weight attributed to accelerations (ACC3). It should be noted that PC2 also included the deceleration variable (DEC3).

**TABLE 3:** Principal component analysis results for external load variables showing eigenvalues, percentage of variance explained, cumulative variance explained, and component loadings for the three principal components.

Principal component (PC)	Eigenvalue	% of total variance explained (VE)	Cumulative % of total VE
1	4.19	34.9	34.9
2	3.90	32.5	67.4
3	2.75	22.9	90.3

**PC**			

**External variables**	**1**	**2**	**3**

TD24 (m)	0.926		
TD21 (m)	0.894		
Vmax (km · h^−1^)	0.889		
TD18 (m)	0.843		
TD14 (m)	0.721		
ALoad (au)		0.904	
DUR (min)		0.880	
TD (m)		0.871	
DEC3 (n)		0.667	0.661
ACC3 (n)			0.912
ACC4 (n)			0.892
DEC4 (n)			0.734

Note: PC = principal component, DUR = session duration, TD = total distance, TD14 = running distance at > 14 km · h^-1^, TD18 = high-speed running distance at > 18 km · h^-1^, TD21 = sprinting distance at > 21 km · h^-1^, TD24 = high sprinting distance at > 24 km · h^-1^, aLoad = acceleration load (arbitrary units, au), Vmax = maximum speed (in km · h^-1^), ACC3 = accelerations > 3 m · s^−2^ (counts), ACC4 = accelerations > 4 m · s^−2^ (counts), DEC3 = decelerations < -3 m · s^−2^ (counts), DEC4 = decelerations < -4 m · s^−2^ (counts).

**FIG. 1:**
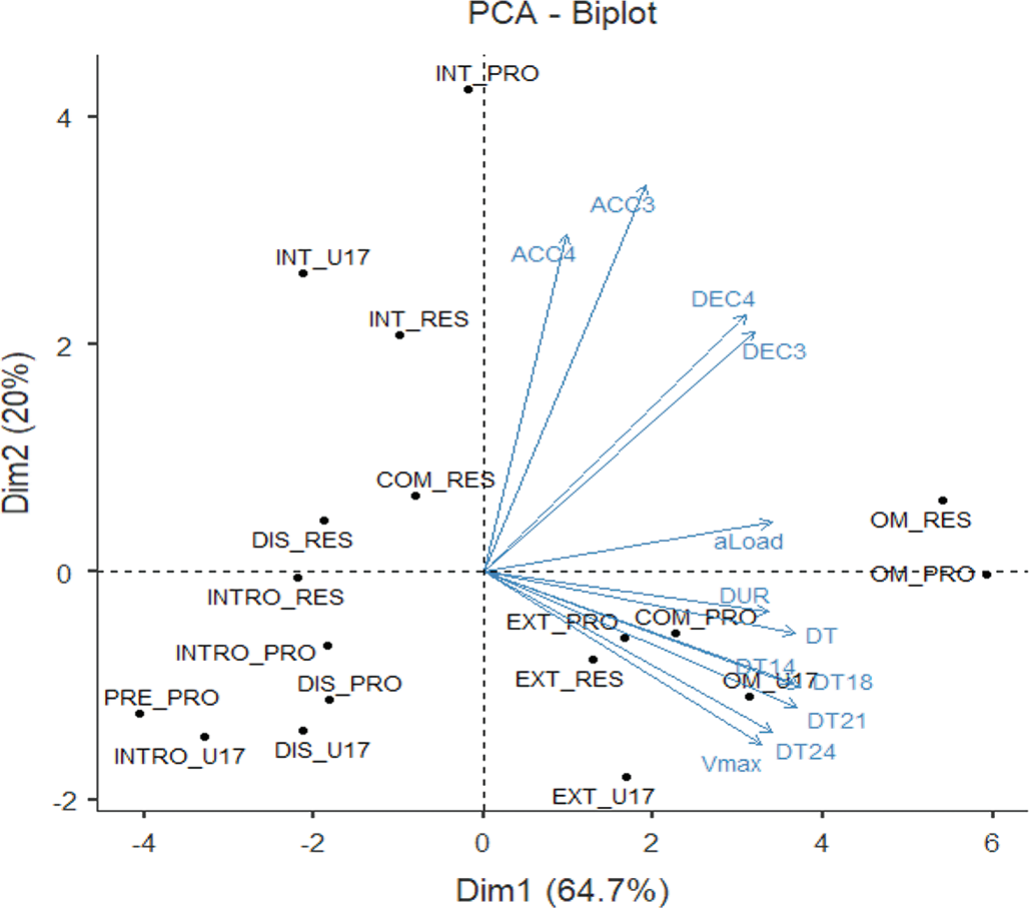
Distribution of training sessions, official matches and external load variables in the two first dimensions of the PCA analysis (PC1 and PC2). PRO = professional team; RES = reserve team and U17 = under-17 team. INTRO = introductory session, EXT = extensive session, INT = intensive session, OM = official match, DIS = discontinuous session, PRE = pre-match session and COMP = compensatory session. DUR = session duration, TD = total distance, TD14 = running distance at > 14 km · h^−1^, TD18 = high-speed running distance at > 18 km · h^−1^, TD21 = sprinting distance at > 21 km · h^−1^, TD24 = high sprinting distance at > 24 km · h^−1^, aLoad = acceleration load (arbitrary units, au), Vmax = maximum speed (in km · h^−1^), ACC3 = accelerations > 3 m · s^−2^ (counts), ACC4 = accelerations > 4 m · s^−2^ (counts), DEC3 = decelerations < -3 m · s^−2^ (counts), DEC4 = decelerations < -4 m · s^−2^ (counts). The category collects the link between session type and team (e.g. OM_RES means the official match of the reserve team).

Figure 1 shows the distribution of training sessions and official matches considering the first two PCs of the PCA analysis (PC1 and PC2), while plotting in blue the vectors representing the magnitude and direction of the variables in these PCs.

**FIG. 2:**
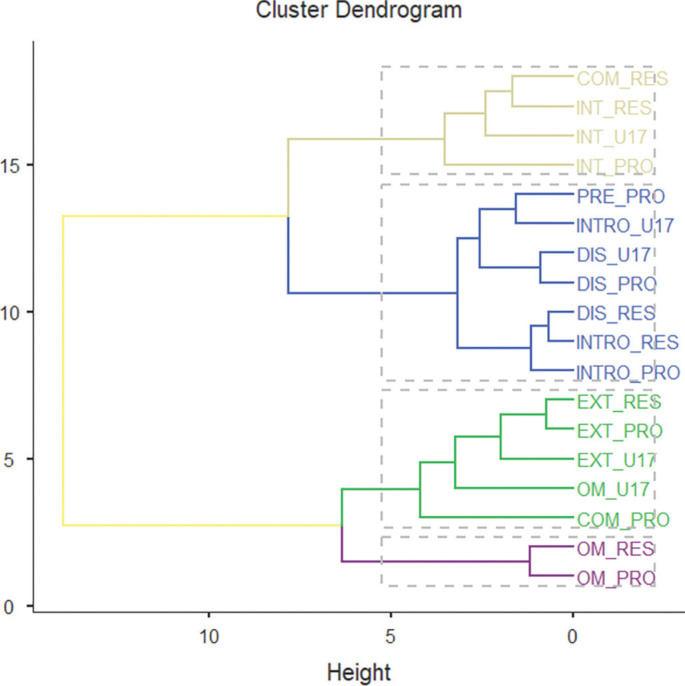
Cluster dendrogram of the training sessions and official matches in the four clusters. PRO = professional team; RES = reserve team and U17 = under-17 team. INTRO = introductory session, EXT = extensive session, INT = intensive session, OM = official match, DIS = discontinuous session, PRE = pre-match session and COMP = compensatory session. The category collects the link between session type and team (e.g., OM_ RES means the official match of the reserve team).

Figure 2 shows the grouping of training sessions and official matches according to the four clusters. The figure is based on the mean data for each variable of the combination of team and type of session. The figure describes how sessions of similar typology were grouped in the same cluster or, at least, distributed in nearby clusters. Generally, it could be said that INT sessions were grouped in one of the clusters, EXT sessions in a different one, OM sessions in another one, and sessions with lower physical demand (e.g., INTRO, DIS, and PRE) were grouped together.

Table 4 presents the average values of each external load variable according to the cluster to which they belong. Cluster 1 represents sessions characterized by lower physical demands across all locomotor and neuromuscular dimensions, including shorter distances covered, fewer acceleration and deceleration efforts, reduced distances at high speeds and lower peak speeds. Cluster 2 comprises sessions with high locomotor demand, characterized by elevated running speeds but lower total distance and moderate neuromuscular demands. Cluster 3 represents the sessions with the highest demands across nearly all the external load variables analysed. Finally, cluster 4 includes sessions with a predominant focus on neuromuscular demand, as indicated by higher values in variables such as ACC3, DEC3, ACC4 and DEC4.

**TABLE 4:** Mean ± SD of the external load variables for each of the four clusters.

Clustering					

External variables	1	2	3	4	Differences (P_tukey_ < 0.05)
DUR (min)	57.0 (± 7.6)	66.5 (± 12.7)	**94.8 (± 3.6)**	61.6 (± 6.8)	3 > 2 = 4 > 1
aLoad (au)	1,547 (± 273.6)	1,996 (± 403.5)	**2,938 (± 135.0)**	2,030 (± 216.8)	3 > 2 = 4 > 1
TD (m)	4,018 (± 913.0)	**6,030 (± 1,687.9)**	**9,811 (± 418.9)**	4,949 (± 595.3)	3 > 2 > 4 > 1
TD14 (m)	441.0 (± 177.6)	**1,067.8 (± 413.7)**	**1,760.3 (± 210.1)**	577.1 (± 214.9)	3 > 2 > 1 = 4
TD18 (m)	94.6 (± 45.9)	**361.2 (± 149.2)**	**589.8 (± 103.3)**	132.2 (± 69.9)	3 > 2 > 1 = 4
TD21 (m)	34.7 (± 26.4)	**179.1 (± 94.1)**	**297.5 (± 71.3)**	39.3 (± 27.5)	3 > 2 > 1 = 4
TD24 (m)	6.4 (± 6.6)	**56.9 (± 45.2)**	**83.4 (± 33.5)**	3.9 (± 4.4)	3 > 2 > 1 = 4
Vmax (km · h^−1^)	22.8 (± 1.3)	**25.9 (± 1.5)**	**26.4 (± 0.7)**	22.9 (± 1.0)	3 = 2 > 1 = 4
ACC3 (n)	22.4 (± 4.9)	29.2 (± 6.2)	**35.9 (± 4.7)**	**42.1 (± 9.2)**	4 > 3 > 2 > 1
DEC3 (n)	29.3 (± 7.6)	39.7 (± 8.0)	**58.5 (± 7.3)**	**51.5 (± 8.7)**	3 > 4 > 2 > 1
ACC4 (n)	2.4 (± 1.0)	3.7 (± 1.9)	**4.0 (± 1.2)**	**5.9 (± 4.0)**	4 > 3 = 2 > 1
DEC4 (n)	6.4 (± 2.2)	9.0 (± 2.6)	**15.3 (± 3.0)**	**13.0 (± 5.4)**	3 = 4 > 2 > 1

Note: 1, 2, 3 and 4 are the clusters. DUR = session duration, TD = total distance, TD14 = running distance at > 14 km · h^−1^, TD18 = high-speed running distance at > 18 km · h^−1^, TD21 = sprinting distance at > 21 km · h^−1^, TD24 = high sprinting distance at > 24 km · h^−1^, aLoad = acceleration load (arbitrary units, au), Vmax = maximum speed (in km · h^−1^), ACC3 = accelerations > 3 m · s^−2^ (counts), ACC4 = accelerations > 4 m · s^−1^ (counts), DEC3 = decelerations < -3 m · s^−1^ (counts), DEC4 = decelerations < -4 m · s^−1^ (counts).

**FIG. 3:**
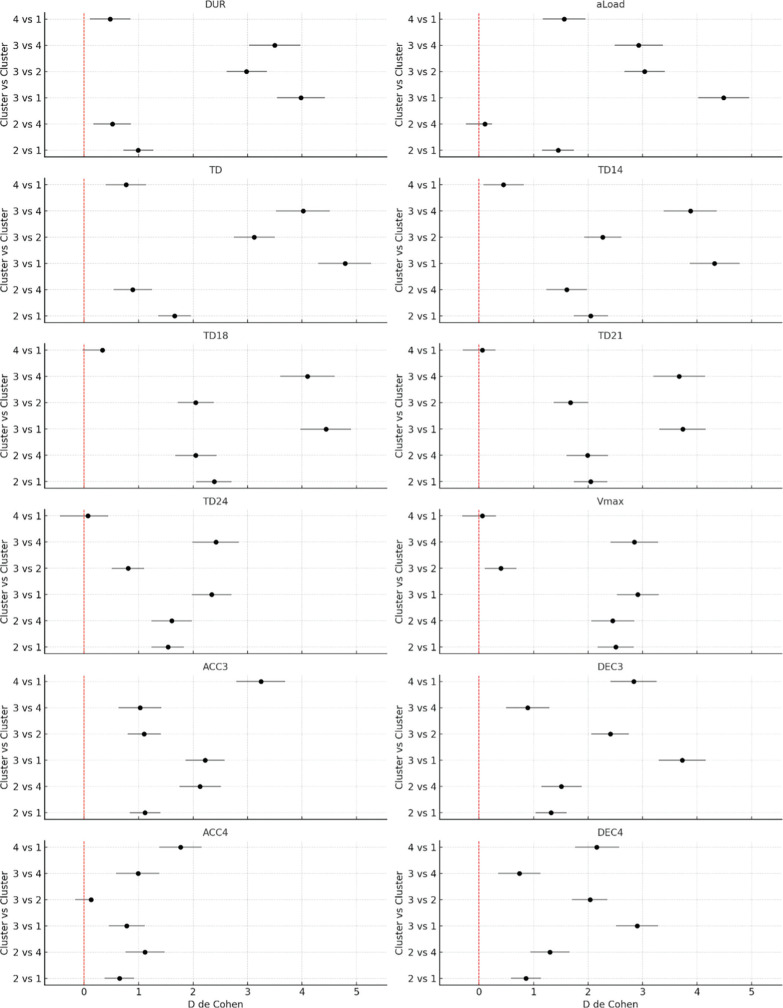
Comparison of Cohen’s d values and confidence intervals (95%CI) between clusters for the variables: DUR = session duration, TD = total distance, TD14 = running distance at > 14 km · h^−1^, TD18 = high-speed running distance at > 18 km · h^−1^, TD21 = sprinting distance at > 21 km · h^−1^, TD24 = high sprinting distance at > 24 km · h^−1^, aLoad = acceleration load (arbitrary units, au), Vmax = maximum speed (in km · h^−1^), ACC3 = accelerations > 3 m · s^−2^ (counts), ACC4 = accelerations > 4 m · s^−2^ (counts), DEC3 = decelerations < -3 m · s^−2^ (counts), DEC4 = decelerations < -4 m · s^−2^ (counts).

Clusters 2 and 3 exhibit the most significant differences compared to other clusters across multiple variables, highlighting distinct performance profiles and physical load characteristics. Notably, variables related to high-speed distances (TD14, TD18 and TD21) and maximum speed (Vmax) show the largest effect sizes (Figure 3), indicating substantial differences between clusters in these measures.

In the comparisons of DUR and aLoad, there are significant differences between the clusters (p < 0.05). For DUR, a very large effect size is observed between cluster 3 and the others, particularly with clusters 1 and 4, indicating that players in cluster 3 have longer duration sessions. aLoad follows a similar pattern, with very large differences between the clusters, especially between cluster 3 and clusters 1 and 4.

For speed variables such as TD14, TD18, TD21, TD24 and Vmax, the results reflect significant differences between clusters (p < 0.05), especially between cluster 3 and the other clusters. TD14 and TD18 show a clear separation between the clusters, with cluster 3 covering significantly more distance at these speeds compared to clusters 1 and 2 (p < 0.05). TD21 and TD24 follow a similar trend, indicating that cluster 3 engages in more high-speed activities, reflecting higher physical demands during training or matches. Vmax also shows differences (p < 0.05), with cluster 3 reaching higher maximum speeds, highlighting superior physical performance in this cluster (Figure 3).

Regarding accelerations (ACC3 and ACC4) and decelerations (DEC3 and DEC4), the results suggest that cluster 4 experiences the highest physical demands in terms of the number of speed changes. In both accelerations greater than 3 m · s^−2^ (ACC3) and 4 m · s^−2^ (ACC4), cluster 4 shows significantly higher values compared to the other clusters (p < 0.05), indicating that players in this type of sessions perform more accelerations. The same pattern is seen for decelerations (DEC3 and DEC4), where cluster 4 also stands out, reflecting a high capacity for rapid pace changes. Cluster 3 also shows large differences compared to clusters 1 and 2, but cluster 4 appears to be subjected to the highest demands in terms of both acceleration and deceleration (Figure 3).

The cluster association assigned to each type of session according to the team studied described a particular grouping of training sessions and matches (Table 5). Cluster analysis identified four distinct session types: low-demand sessions (including introductory, pre-match, and discontinuous training sessions), moderate-demand sessions characterized by high-speed (such as compensatory and extensive training sessions), sessions with the highest overall demands (official matches), and sessions with high neuromuscular demands (INT, intensive training sessions). The majority of COM sessions were grouped in cluster 2 for both PRO (86%) and RES (83%). For DIS sessions, teams show different distributions. PRO groups these sessions in cluster 2 (68%), while RES has a higher proportion in cluster 1 (55%), followed by cluster 4 (32%). U17 groups this type of DIS sessions between clusters 1 (48%) and 2 (52%). In EXT sessions, practically all three teams (PRO, RES, and U17) grouped their sessions in cluster 2 (94%–96%). The INT sessions show a wider distribution; although the PRO and RES teams have their sessions mostly located in cluster 4 (81% and 71%, respectively), while U17 grouped almost entirely in cluster 1 (80%). In the INTRO sessions, PRO, RES, and U17 team have a clustering in cluster 1 (57%–83%), with smaller proportions in cluster 2 and somewhat in cluster 4 for PRO and RES. Finally, all of the PRO and RES OM were grouped in cluster 3 (100%), while U17 were distributed between clusters 2 (60%) and 3 (40%).

**TABLE 5:** Distribution of session types and all teams in relation to the four clusters.

Clustering						

Type of session	team	1	2	3	4	Total
COM	PRO	14 %	**86 %**	0 %	0 %	7
	RES	8 %	**83 %**	0 %	8 %	12
	U17					
	Total	2	16	0	1	19

DIS	PRO	27 %	**68 %**	0 %	5 %	22
	RES	**55 %**	14 %	0 %	32 %	22
	U17	**48 %**	**52 %**	0 %	0 %	23
	Total	29	30	0	8	67

EXT	PRO	0 %	**94 %**	6 %	0 %	17
	RES	4 %	**96 %**	0 %	0 %	23
	U17	0 %	**95 %**	5 %	0 %	21
	Total	1	58	2	0	61

INT	PRO	6 %	13 %	0 %	**81 %**	16
	RES	17 %	13 %	0 %	**71 %**	24
	U17	**80 %**	20 %	0 %	0 %	5
	Total	9	6	0	30	45

INTRO	PRO	**57 %**	36 %	0 %	7 %	14
	RES	**67 %**	17 %	0 %	17 %	12
	U17	**83 %**	17 %	0 %	0 %	12
	Total	26	9	0	3	38

OM	PRO	0 %	0 %	**100 %**	0 %	28
	RES	0 %	0 %	**100 %**	0 %	27
	U17	0 %	**60 %**	**40 %**	0 %	30
	Total	0	18	67	0	85

PRE	PRO	**100 %**	0 %	0 %	0 %	21
	RES					
	U17					
	Total	21	0	0	0	21

Total	PRO	30 %	35 %	23 %	12 %	125
	RES	22 %	33 %	23 %	23 %	120
	U17	27 %	58 %	14 %	0 %	91
	Total	88	137	69	42	336

Note: 1, 2, 3, and 4 are the clusters. PRO = professional team; RES = reserve team and U17 = under-17 team. INTRO = introductory session, EXT = extensive session, INT = intensive session, OM = official match, DIS = discontinuous session, PRE = pre-match session, COM = compensatory session. U17 did not have a pre-match session.

## DISCUSSION

This study mainly aimed to investigate the external load demands placed on female football players during training sessions and official matches, focusing on differences across various levels of competition and types of training sessions. It also represents the first application of cluster analysis to comprehensively characterize women’s football training microcycles in relation to match demands. The findings of this study provide a comprehensive perspective on the physical demands placed on female football players during training sessions and official matches, highlighting that external load can be reduced to three PCs and that training sessions can be grouped into four distinct clusters in relation to match demands. These findings represent the main contribution of the study and offer a novel framework for understanding women’s football microcycles. By addressing these gaps, clubs can better support their players’ long-term development and performance, ensuring a smoother transition across developmental stages. At the same time, this framework is crucial for optimizing training programs, enhancing performance, and mitigating injury risk in a rapidly evolving sport.

The study identified key patterns in external load variables and session types: 1) Twelve external load variables were grouped into three components—high-speed variables, volume (e.g., duration, total distance, and decelerations), and changes in speed (accelerations and decelerations). 2) Four session types emerged from cluster analysis: low-demand sessions (introductory, pre-match, and discontinuous training sessions), high-speed but moderate-demand sessions (compensatory and extensive training sessions), highest-demand sessions (official matches), and high-neuromuscular-demand sessions (intensive training sessions). 3) Clusters 2 (compensatory and extensive training sessions) and 3 (official matches) showed significant differences, particularly in high-speed distances and maximum speed, highlighting distinct performance profiles. 4) Teams generally distributed sessions consistently across clusters, except for U17 during intensive and official matches, and the PRO team during discontinuous sessions, which varied by team analysed.

The PCA shows that three main components collectively represent over 90% of the information. The first component comprises the variables most associated with high-speed; the second component includes the variables most associated with training volume (e.g., duration, total distance, and decelerations), and the third component relates to changes in speed (accelerations and decelerations). However, the relative contribution of each component may have been influenced by contextual aspects such as differences in weekly structures across squads, match exposure, or the limited number of official matches analysed. While these factors should be acknowledged as limitations, the use of PCA provided a robust reduction of dimensionality and revealed consistent patterns despite such variability. This analysis indicates that to represent the external load of training, we must conduct a multivariable analysis that considers the three dimensions identified in the PCA, as previously observed in studies conducted in men’s football [21, 32, 33]. Specifically, Casamichana et al. [21] and Zurutuza et al. [33] highlighted the need to combine complementary variables to explain training load variability, while Teixeira et al. [32] demonstrated, through a PCA approach in youth male players, that three main components (volume, high-speed activity, and accelerations/decelerations) summarize the majority of training load. The present results in female players align with these findings, confirming that external load in women’s football is also multidimensional, while representing the first formal application of PCA in this context.

The clustering analysis revealed clear distinctions in the external load demands of various session types. Sessions with the highest physical demands (cluster 3), characterized by total distance, high-speed running, and sprinting metrics, were predominantly associated with OM for the PRO and RES teams. This aligns with previous research identifying matches as the most physically intensive activity in football [1, 15], and suggests that cluster 3 can be strategically used as an overload stimulus to enhance aerobic and sprint capacity. Similarly, high neuromuscular demand (cluster 4), driven by variables such as accelerations and decelerations, was primarily linked to intensive training sessions. This suggests that high-intensity training is intentionally structured to mimic the physical and tactical challenges of competitive matches, particularly for higher-tier teams [34]. In contrast, cluster 1 grouped the lowest-demand sessions (e.g., INTRO, DIS or PRE), which are valuable for managing residual fatigue and pre-match tapering, whereas cluster 2 presented profiles close to match intensity but with lower total volume, making it useful to maintain competition-level exposure while limiting accumulated fatigue. These findings are consistent with recent evidence showing that weekly load distribution in female professional teams is strongly influenced by match scheduling [35], reinforcing the value of using cluster analysis to better understand microcycle organization and contextual variability. In this regard, previous work has described how professional teams often apply an inverted-U distribution of training loads within the microcycle, featuring horizontal alternation across the middle days to stimulate the three primary physical qualities (strength, endurance, and speed), while reserving the days immediately prior to competition for activation and tapering phases [36, 37]. Together, these profiles provide coaches with a practical framework to distribute training stimuli across the microcycle and balance load and recovery.

When analysing how these clusters were distributed across squads, clear differences emerged. U17 players accumulated a higher proportion of low-demand sessions (cluster 1), with INTRO and DIS being dominant, reflecting reduced exposure to high-speed and neuromuscular loads compared to PRO and RES. This observation complements recent evidence showing that female players selected for national teams exhibit superior sprinting and acceleration capabilities compared to non-selected players, highlighting that physical qualities are a key determinant of competitive progression [38]. This finding is consistent with recent evidence in women’s football reporting substantial external load differences between U17 and senior players, particularly in sprinting and accelerations [39], and highlights the importance of designing progressive training strategies tailored to female developmental trajectories. In contrast, PRO and RES teams exhibited a higher proportion of highdemand sessions, which provides greater exposure to match-like physical demands. While EXT sessions showed some consistency across squads, clustering analysis also identified discrepancies: for example, DIS sessions were assigned to cluster 2 for PRO but to clusters 1 and 4 for RES, and OM sessions in U17 were grouped with EXT and COMP in cluster 2 rather than with the OM of PRO and RES in cluster 3. This could be partly explained by the lower physical performance level of the U17 players, who may have difficulty reaching absolute thresholds for intense actions such as accelerations > 4 m · s^−2^ [32]. In this regard, adopting thresholds relative to the players’ individual maximum capacity could make external load data more comparable across squads [40, 41]. Taken together, these inconsistencies suggest that training load distribution within the same club is not fully harmonized, potentially compromising the smooth progression of players from U17 to senior squads. To mitigate this, clubs should seek to align training principles across categories, while still accounting for developmental differences, so that players are gradually exposed to workloads that prepare them for the physical and tactical demands of professional football.

Despite its contributions, this study is not without limitations. The analysis focused exclusively on external load variables and did not consider internal load measures such as heart rate or subjective perceived exertion, which could provide a more comprehensive understanding of training and official match demands. Furthermore, relativizing the external load variables based on players’ individual maximum capacities would likely have enhanced comparability across competitive levels. Similarly, expressing training demands as a percentage of each team’s own match reference could have provided a fairer comparison between squads. Without this approach, U17 players may appear underestimated in the present results, not because their training sessions are less intense, but because comparisons were made using absolute values rather than relative to their match context. In addition, future research should incorporate contextual factors such as effective playing time and variability in microcycle length, as recent evidence in professional female players has demonstrated that differences in microcycle duration significantly impact both external and internal load measures [42]. Addressing these aspects would improve the ecological validity, comparability, and generalizability of future research in women’s football

## CONCLUSIONS

This study underscores the importance of a structured and coherent training load distribution across session types and teams in elite women’s football. The findings highlight areas for optimization, particularly in aligning the training demands of developmental teams with the physical and tactical requirements of professional football. By addressing these gaps, clubs can better support their players’ long-term development and performance, ensuring a smoother transition across developmental stages.

Future research should build on these findings by integrating internal load measures (e.g., heart rate, RPE) with external load profiles, and by exploring the longitudinal effects of different training loads on player development, performance, and injury risk. Such approaches would provide a more comprehensive understanding of women’s football microcycles and their impact across competitive levels.

The main practical applications of this study relate to the fact that, despite analysing six different session types, the multivariable analysis of external load identifies four groups of sessions or clusters: low-demand sessions (introductory, pre-match, and discontinuous training sessions); high-speed but moderate-demand sessions (compensatory and extensive training session); highest-demand sessions (official matches); and high-neuromuscular-demand sessions (intensive training sessions). This framework provides coaches and strength and conditioning staff with a practical tool to periodize training loads across the microcycle, optimize the balance between overload and recovery, and progressively prepare young players for the demands of senior football. Such applications are consistent with the inverted-U organization of the microcycle, where horizontal alternation across midweek sessions stimulates complementary physical qualities, while pre-match days are reserved for activation and tapering. By applying these insights, practitioners can enhance performance while reducing the risk of under- or overexposure to specific physical demands.

## References

[1] Griffin J, Newans T, Horan S, Keogh J, Andreatta M, Minahan C. Acceleration and high-speed running profiles of women’s international and domestic football matches. Front Sports Act Living. 2021; 3:604605. doi: 10.3389/fspor.2021.604605. 33842879 PMC8027246

[2] Teixeira JE, Forte P, Ferraz R, Leal M, Ribeiro J, Silva AJ, et al. Monitoring accumulated training and match load in football: a systematic review. Int J Environ Res Public Health. 2021; 18(8):3906. doi: 10.3390/ijerph18083906. 33917802 PMC8068156

[3] FIFA. Women’s football: member associations survey report 2023. Zurich: FIFA; 2023.

[4] Bradley PS, Dellal A, Mohr M, Castellano J, Wilkie A. Gender differences in match performance characteristics of soccer players competing in the UEFA Champions League. Hum Mov Sci. 2014; 33:159–171. 24139663 10.1016/j.humov.2013.07.024

[5] Farhani Z, Amara S, Ben Aissa M, Guelmami N, Bouassida A, Dergaa I. The variability of physical enjoyment, physiological responses, and technical-tactical performance according to the bout duration of small-sided games: a comparative study between female and male soccer players. BMC Sports Sci Med Rehabil. 2024; 16:39. 38570786 10.1186/s13102-023-00794-1 PMC10988879

[6] Okholm Kryger K, Wang A, Mehta R, Impellizzeri FM, Massey A, McCall A. Research on women’s football: a scoping review. Sci Med Football. 2022; 6(5):549–558. 10.1080/24733938.2020.1868560 36540910

[7] Oliveira R, Martins A, Moreno-Villanueva A, Brito JP, Nalha M, Rico-González M, et al. Reference values for external and internal training intensity monitoring in professional male soccer players: a systematic review. Int J Sports Sci Coach. 2022; 17(6):1506–1530.

[8] Martin-García A, Gómez Díaz A, Bradley PS, Morera F, Casamichana D. Quantification of a professional football team’s external load using a microcycle structure. J Strength Cond Res. 2018; 32(12):3511–3518. 30199452 10.1519/JSC.0000000000002816

[9] Gomez-Villaseca M, Rehbein C, Earp J, Ramirez-Campillo R, Penailillo L. Match-related activity profiles of youth female and male football players during World Cup classification tournaments and their relationship to body composition. J Sports Med Phys Fitness. 2024; 64(8):xxxxߝxxxx. 10.23736/S0022-4707.24.15441-2 38841727

[10] Cardoso de Araújo M, Baumgart C, Jansen CT, Freiwald J, Hoppe MW. Sex differences in physical capacities of German Bundesliga soccer players. J Strength Cond Res. 2020; 34(8):2329–2337. 29927885 10.1519/JSC.0000000000002662

[11] Scott D, Bruinvels G, Norris D, Lovell R. The dose–response in elite soccer: preliminary insights from menstrual-cycle tracking during the FIFA Women’s World Cup 2019. Int J Sports Physiol Perform. 2024; 19(4):331–339. 38198788 10.1123/ijspp.2022-0282

[12] Carmichael MA, Thomson RL, Moran LJ, Wycherley TP. The impact of menstrual cycle phase on athletes’ performance: a narrative review. Int J Environ Res Public Health. 2021; 18(4):1667. 33572406 10.3390/ijerph18041667 PMC7916245

[13] Colenso-Semple LM, D’Souza AC, Elliott-Sale KJ, Phillips SM. Current evidence shows no influence of women’s menstrual cycle phase on acute strength performance or adaptations to resistance exercise training. Front Sports Act Living. 2023; 5:1054542. 37033884 10.3389/fspor.2023.1054542 PMC10076834

[14] Delgado-Bordonau JL, Mendez-Villanueva A. Tactical periodization. Soccer J. 2012; (June):28–34.

[15] Guridi I, Castellano J, Echeazarra I. Physical demands and internal response in football sessions according to tactical periodization. Retos. 2021; 41:1–7. 10.1123/ijspp.2019-0829 33626511

[16] Olaizola A, Errekagorri I, Lopez-de-Ipina K, Calvo PM, Castellano J. Comparison of the external load in training sessions and official matches in female football: a case report. Int J Environ Res Public Health. 2022; 19(23):15820. 36497893 10.3390/ijerph192315820 PMC9736486

[17] Soler-López A, Gómez-Carmona CD, Moreno-Villanueva A, Gutiérrez AM, Pino-Ortega J. Effects of congested matches and training schedules on salivary markers in elite futsal players. Appl Sci. 2024; 14(12):5478.

[18] Soler-López A, Moreno-Villanueva A, Gómez-Carmona CD, Pino-Ortega J. The role of biomarkers in monitoring chronic fatigue among male professional team athletes: a systematic review. Sensors. 2024; 24(21):6591. 39517758 10.3390/s24216862 PMC11548435

[19] Zech A, Hollander K, Junge A, Steib S, Groll A, Heiner J, et al. Sex differences in injury rates in team-sport athletes: a systematic review and meta-regression analysis. J Sport Health Sci. 2022; 11(1):104–114. 34052518 10.1016/j.jshs.2021.04.003 PMC8847930

[20] Rojas-Valverde D, Pino-Ortega J, Gómez-Carmona CD, Rico-González M. A systematic review of methods and criteria standard proposal for the use of principal component analysis in team’s sports science. Int J Environ Res Public Health. 2020; 17(23):8712. 33255212 10.3390/ijerph17238712 PMC7727687

[21] Casamichana D, Castellano J, Gómez Díaz A, Martín-García A. Looking for complementary intensity variables in different training games in football. J Strength Cond Res. 2019; 33(11):3080–3087. 10.1519/JSC.0000000000003025 30844980

[22] Weaving D, Jones B, Marshall P, Till K, Abt G. Multiple measures are needed to quantify training loads in professional rugby league. Int J Sports Med. 2017; 38(10):735–740. 28783849 10.1055/s-0043-114007

[23] Casal CA, Losada JL, Barreira D. Multivariate exploratory comparative analysis of LaLiga teams: principal component analysis. Int J Perform Anal Sport. 2021:xxxx–xxxx. 10.3390/ijerph18063176 PMC8003572 33808634

[24] Rodríguez Porras LD, Solano-Mora L, Rivas-Borbón M, Moreno-Villanueva A, Soler-López A, Pino-Ortega J, et al. Profile of physical demands in female soccer players during competitions: a systematic review. Strength Cond J. 2024; 46(5):567–586.

[25] Delaney JA, Cummins CJ, Thornton HR, Duthie GM. Importance, reliability, and usefulness of acceleration measures in team sports. J Strength Cond Res. 2018; 32(12):3485–3493. 28195980 10.1519/JSC.0000000000001849

[26] Weaving D, Beggs C, Dalton-Barron N, Jones B, Abt G. Visualizing the complexity of the athlete-monitoring cycle through principal-component analysis. Int J Sports Physiol Perform. 2019; 14(9):1304–1310. 31569072 10.1123/ijspp.2019-0045

[27] Kaiser HF. The application of electronic computers to factor analysis. Educ Psychol Meas. 1960; 20(1):141–151.

[28] Williams S, Trewartha G, Cross MJ, Kemp SPT, Stokes KA. Monitoring what matters: a systematic process for selecting training-load measures. Int J Sports Physiol Perform. 2017; 12:101–106. 10.1123/ijspp.2016-0337 27834553

[29] Weaving D, Marshall P, Earle K, Nevill A, Abt G. Combining internal- and external-training-load measures in professional rugby league. Int J Sports Physiol Perform. 2014; 9(6):905–912. 24589469 10.1123/ijspp.2013-0444

[30] Kodinariya TM, Makwana PR. Review on determining number of cluster in K-means clustering. Int J Adv Res Comput Sci Manag Stud. 2013; 1(6):90–95.

[31] Hopkins WG, Marshall SW, Batterham AM, Hanin J. Progressive statistics for studies in sports medicine and exercise science. Med Sci Sports Exerc. 2009; 41(1):3–12. 19092709 10.1249/MSS.0b013e31818cb278

[32] Teixeira JE, Forte P, Ferraz R, Branquinho L, Morgans R, Silva AJ, et al. Resultant equations for training load monitoring during a standard microcycle in sub-elite youth football: a principal components approach. PeerJ. 2023; 11:e14785. 37554335 10.7717/peerj.15806 PMC10405799

[33] Zurutuza U, Castellano J, Echeazarra I, Guridi I, Casamichana D. Selecting training-load measures to explain variability in football training games. Front Psychol. 2020; 11:2089. 32038349 10.3389/fpsyg.2019.02897 PMC6992576

[34] Mohr M, Krustrup P, Bangsbo J. Fatigue in soccer: a brief review. J Sports Sci. 2005; 23(6):593–599. 16195008 10.1080/02640410400021286

[35] Scanlan AT, Miller D, Lundquist M, Elsworthy N, Lastella M. Load distribution across weekly microcycles according to match schedule in a team competing in the Australian national A-League Women’s soccer competition. Biol Sport. 2025; 42(2):265–277. 40182733 10.5114/biolsport.2025.144413 PMC11963116

[36] Buchheit M, Lacome M, Cholley Y, Simpson BM. Neuromuscular responses to conditioned soccer sessions assessed via GPS-embedded accelerometers: insights into tactical periodization. Int J Sports Physiol Perform. 2018; 13(5):577–583. 28872370 10.1123/ijspp.2017-0045

[37] Marín K, Castellano J. Comparison of different coach competition micro-cycle planning strategies in professional soccer. Sustainability. 2023; 15:16218.

[38] Savolainen E, Ihalainen J, Weckström K, Vänttinen T, Walker S. Female football players’ key physical qualities: playing-position specific comparison between national-team selected and non-selected players. Biol Sport. 2025; 42(3):109–117. 40657005 10.5114/biolsport.2025.146780 PMC12244401

[39] Mazola I, Valdés M, Romero-Moraleda B, González-García J. From youth to senior: external load progression and positional differences in Spanish women’s national teams during World Cup competitions. Appl Sci. 2025; 15:8421.

[40] Clemente F, Ramirez-Campillo R, Beato M, Moran J, Kawczynski A, Makar P, et al. Arbitrary absolute vs. individualized running speed thresholds in team sports: a scoping review with evidence gap map. Biol Sport. 2023; 40(3):919–943. 37398971 10.5114/biolsport.2023.122480 PMC10286616

[41] Harkness-Armstrong A, Till K, Datson N, Myhill N, Emmonds S. A systematic review of match-play characteristics in women’s soccer. PLoS ONE. 2022; 17(4):e0268334. 35771861 10.1371/journal.pone.0268334 PMC9246157

[42] Posse-Álvarez M, Solleiro-Duran D, Lorenzo-Martínez M, Iglesias-Soler E, Oliva-Lozano JM, Padrón-Cabo A. Does microcycle length influence the external and internal load in professional female soccer players? Biol Sport. 2025; 42(2):215–223. 40182727 10.5114/biolsport.2025.144408 PMC11963138

